# A Novel Fault-Tolerant Information Fusion Method for Integrated Navigation Systems Based on Fuzzy Inference

**DOI:** 10.3390/s25051624

**Published:** 2025-03-06

**Authors:** Yixian Zhu, Minmin Zhang, Ling Zhou, Ting Cai

**Affiliations:** 1 School of Mechanical Engineering, Nantong University, Nantong 226019, China; caiting@ntu.edu.cn; 2 China Construction Second Engineering Bureau Company, Beijing 101101, China; mmzhang824@163.com; 3 School of Physics and Electronic Engineering, Yuncheng University, Yuncheng 044000, China; s03031212@126.com

**Keywords:** integrated navigation, information fusion, federated filter, fault-tolerant, fuzzy inference

## Abstract

To enhance the precision and reliability of integrated navigation systems, a novel fault-tolerant information fusion algorithm based on a federated filter is proposed. Decentralized filtering architecture is employed to fuse information from different navigation subsystems. The chi-square detection function and the filter innovation correlation are used as inputs to the fuzzy system, which then outputs the observation quality factor. The observation quality factor directly reflects the reliability of the measurement data and is utilized to adjust the local filter gain matrix online. Additionally, the information sharing coefficients, determined by the observation quality factors, ensure dependable fault isolation while improving the sensitivity of fault detection to gradual faults. Comparative experimental results demonstrate that the proposed method effectively detects various faults and significantly enhances the performance of the integrated navigation system during malfunctions.

## 1. Introduction

Navigation systems, which deliver comprehensive data such as position, speed, and attitude information, are indispensable components of autonomous vehicles [1,2,3]. Among these, the strapdown inertial navigation system (SINS) is the most widely used due to its independence and high sampling frequency. However, SINS suffers from accumulating navigation errors over time caused by the drifts of gyros and accelerometers. To mitigate this issue, auxiliary navigation sensors or systems are introduced to correct SINS errors. Different navigation sensors or systems provide various types of information. For instance, a magnetic compass (MCP) and an altimeter offer heading and height information, respectively. Speed information can be obtained from a Doppler velocity log (DVL) or an odometer (OD). Position data are typically provided by a global positioning system (GPS) or terrain-aided navigation (TAN). These complementary navigation sensors or systems enhance the robustness and environmental adaptability of the navigation system. An integrated navigation system, which combines two or more navigation sensors or subsystems, is designed to fuse information from diverse sources. By integrating the data, such systems provide more accurate and reliable navigation results, ensuring improved performance under varying conditions.

Information fusion algorithms can be classified into three categories: optimization-based, learning-based, and filter-based methods. Optimization-based methods, using Bayesian graph networks, transform the fusion problem into the calculation of the maximum a posteriori estimation of the system state over time [4,5]. Learning-based methods excel at handling highly nonlinear problems, especially when there is no accurate mathematical model available for the sensors [6,7,8]. Currently, the most developed approach, filter-based methods, utilizes a series of noisy measurements to estimate the system state. Filter-based methods can be further divided into centralized and distributed approaches based on the structure of fusion integration. A centralized filter processes information from all subsystems using a single filter [9]. While it can theoretically provide the optimal estimation of the system state, it has two notable drawbacks: high computational cost and poor fault tolerance. The real-time performance of a centralized filter deteriorates as computation grows cubically with the filter dimension. Moreover, due to the strong coupling of subsystems, a failure in one subsystem can negatively affect the entire system. In response to the growing need for parallel processing and enhanced fault tolerance, distributed filtering has gained significant traction. A notable example is the federated filter, which operates based on rigorous information sharing principles [10]. The federated filter offers the advantages of flexible design, reduced computational load, and robust fault tolerance.

As sensor technology advances, more sensors can be integrated into navigation systems, increasing their overall capabilities but also adding complexity. However, the sensors or subsystems used for navigation may occasionally malfunction or fail. For instance, GPS signals can be temporarily lost in urban canyons or tunnels. Similarly, the DVL becomes ineffective when the distance to the underwater floor exceeds its maximum range. Additionally, the geomagnetic field in certain geographical regions can disrupt the normal functioning of MCP. Faulty sensors or subsystems can lead to inaccurate measurements, ultimately impacting the accuracy of integrated navigation results. Therefore, an efficient fault-tolerant strategy, incorporating both fault detection and fault isolation, is essential to ensure reliable system performance.

In conventional fault-tolerant integrated navigation systems, faulty subsystems are completely isolated at the moment a fault is detected. Fault detection results are binary, with only two possible states: fault and non-fault. Correspondingly, the subsystem operates in one of two states: fusion or isolation, with no transitional states in between. The decision to isolate a subsystem relies solely on fault detection results. However, these direct isolation methods overlook the initial effects of gradual faults on the system. Gradual faults are notoriously challenging to detect due to their long detection lag times. As a result, researchers have increasingly focused on developing faster detection methods to reduce this delay [11,12,13,14,15,16]. Liu et al. [11] found that traditional fault detection methods struggle to detect gradual faults, particularly in underwater navigation systems. These types of faults are difficult to identify early, and current techniques often fail to respond in time. This results in inaccurate measurements and degraded system performance before faults are detected. They proposed an enhanced residual chi-square detection method that uses the normalized residual mean and the sum of absolute residuals as criteria to detect gradual faults more promptly. Ref. [12] highlights that traditional fault detection methods struggle to keep up with the dynamic and diverse data from UAV navigation sensors. These methods tend to be too slow and often fail to adapt to rapidly changing conditions, leading to delayed fault detection. Then an adaptive neuro-fuzzy inference system (ANFIS) combined with an online data training mechanism was designed to detect navigation sensor faults in UAVs. Ref. [13] revealed that integrating multiple sensors into a navigation system increases the complexity of fault detection, especially when faults develop gradually. Current methods fail to manage sensor conflicts and mismatched data effectively, which can lead to inaccuracies in navigation. A two-stage fault detection structure is presented in [13], where abrupt and gradual faults are detected using separate modules. The pre-fault detection module employs the sequential probability ratio test (SPRT), while the secondary-fault detection module applies the residual chi-square test.

In contrast, certain methods achieve fault isolation by adjusting the utilization of measurement information based on specific principles. These methods address scenarios where initial faults are minor and go undetected, avoiding the binary classification of the system’s operating state. Instead, fault isolation is realized through the use of fault detection quantitative values. Consequently, determining the relationship between the utilization of measurement information and fault detection parameters is critical for achieving effective fault tolerance. Some fault-tolerant solutions are implemented by optimizing the filter, as the adjustment of measurement information is carried out at the filter level. For example, Gao et al. [17] proposed an adaptive fault-tolerant cubature Kalman filter designed to handle outliers and noise uncertainties in observations. This method employs the chi-square test and sequential probability ratio test to compute the filter gain. In [18], an adaptive sensor fusion technique enhances fault tolerance in integrated navigation systems by using innovation covariance discrepancies to adaptively modify the measurement noise covariance matrix. Liang et al. [19] found that the nonlinear quaternion-based approach to fault tolerance was more efficient in preserving navigation accuracy than traditional linear methods, particularly when applied to real-world UAV systems. They developed fault-tolerant versions of the extended Kalman filter and the divided difference Kalman filter to maintain navigation accuracy in the presence of faults. This approach enlarges the relevant covariance matrices of the projected errors using fault accommodation factors, enabling normal treatment of defective states or measurement components. Additionally, Ref. [20] found that a multi-layer approach to fault tolerance provided greater robustness in high-risk environments. They propose a multi-layer, robust fault-tolerant filtering algorithm for aeronautical vehicles. By implementing this multi-layer strategy, the system could effectively identify and isolate faults at different levels of the navigation system, offering better protection against complex faults.

This paper proposes a fault-tolerant information fusion algorithm based on a variable proportion federated filter structure. To enhance sensitivity to gradual faults, observation quality factors are defined using fuzzy inference. These factors are used to adjust the local filter gains and determine the information sharing coefficients, as they effectively evaluate the accuracy of measurement information. Fault isolation is achieved by adaptively modifying the local filter gains in real time, enabling the system to adjust the use of measurement information dynamically. Furthermore, to improve the accuracy of the global state estimate, variable information sharing coefficients are defined based on the observation quality factors.

The main contributions and innovations of this paper can be summarized as follows:

(1) Fuzzy inference is employed to map the filter innovation to the observational quality factor, enabling more sensitive detection of gradual faults.
(2) The use of variable information sharing coefficients not only enhances the precision of the local filter but also improves the sensitivity of fault detection, particularly for gradual faults.
(3) The proposed adaptive information fusion algorithm mitigates the impact of faults and significantly enhances the fault tolerance of integrated navigation systems.

The paper is organized as follows. The federated filter model for fault-tolerant integrated navigation systems is described in Section 2. Section 3 presents the fault-tolerant information fusion algorithm based on fuzzy inference. The simulation results and analysis are provided in Section 4. Finally, Section 5 is devoted to the conclusion.

## 2. Federated Filter Based Fault-Tolerant Navigation System

### 2.1. System Structure

**Figure 1:**
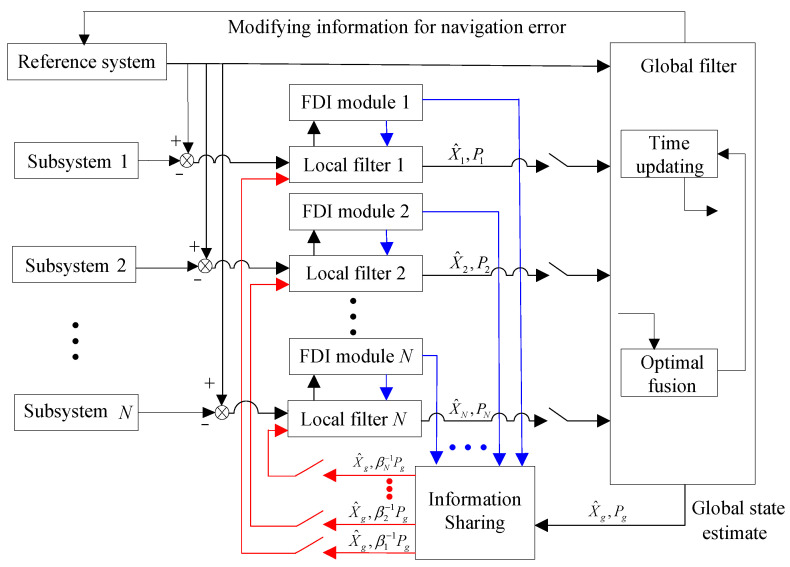
Fault-tolerant integrated navigation structure based on federated filter.

To enhance fault tolerance, a federated filter with a conditional reset scheme for information sharing is implemented for integrated navigation, as shown in Figure 1. The SINS is generally used as the reference system due to its high independence and reliability, and its computations can provide comprehensive navigation information, including position, velocity, and attitude data. However, because the navigation errors of SINS accumulate rapidly over time, it requires auxiliary navigation systems to correct these errors. The auxiliary navigation subsystems can include OD, GPS, MCP, or DVL, among others, and one or more of these can be selected to form an integrated navigation system with SINS. Specifically, OD provides velocity information, GPS provides position information, MCP provides heading information, and DVL provides velocity information. The local filter processes the difference between the measurement data from the subsystem and the reference system. An improved Kalman filter is used to execute the local filter, with its gain updated in real time based on the quality of the measurement information. Each local filter is equipped with fault detection and isolation (FDI) modules to detect faults within the subsystem and isolate the faulty data. The FDI module uses fuzzy inference to determine the presence and degree of faults and subsequently provides the corresponding observational quality factor value. Faulty measurement information is isolated by adjusting the local filter’s gain matrix based on the observation quality factor. Simultaneously, the reconstruction of observation quality factors determines the information sharing coefficients. The global state estimates are fed back into each local filter to further optimize the local filter state estimates. By feeding global information back into the local filters, higher-level information sharing is realized, which improves the robustness of the local filters. The global filter’s state estimate helps enhance the local filters’ handling of uncertainty and improves system-wide performance by enhancing data consistency. Additionally, the global filter resets the estimation covariance matrices of the local filters using the information sharing coefficients to ensure effective fault isolation. Ultimately, the global state estimate output by the global filter is fed back to the reference system to correct its calculation errors, thereby obtaining accurate navigation results. This federated filter structure is designed to maximize fusion accuracy while ensuring the system’s fault tolerance, offering a robust solution for integrated navigation systems.

### 2.2. System Model

Based on the analysis of INS error sources, the errors in INS navigation parameters are selected as the state variables for the integrated navigation system [21,22]. The east-north-up (ENU) coordinate system is adopted as the navigation frame, while the right-front-up (RFU) coordinate system is chosen as the body frame. Accordingly, the 15-dimensional state vector is defined as follows:

(1) $$X_{k}={\left[\;\phi_{e}\;\;\;\;\;\phi_{n}\;\;\;\;\;\phi_{u}\;\;\;\;\;\delta V_{e}\;\;\;\;\;\delta V_{n}\;\;\;\;\;\delta V_{u}\;\;\;\;\;\delta L\;\;\;\;\;\delta \lambda \;\;\;\;\;\delta h\;\;\;\;\;\;\varepsilon_{x}\;\;\;\;\;\varepsilon_{y}\;\;\;\;\;\varepsilon_{z}\;\;\;\;\nabla_{x}\;\;\;\;\nabla_{y}\;\;\;\;\nabla_{z}\right]}^{T}$$

where *ϕ_e_*, *ϕ_n_*, and *ϕ_u_* are attitude errors in the navigation frame in the east, north, and up directions, respectively. *δV_e_*, *δV_n_*, and *δV_u_* are velocity errors in the east, north, and up directions, respectively. *δL*, *δλ*, and *δh* are latitude error, longitude error, and height error, respectively. *ε_x_*, *ε_y_*, *ε_z_* are gyroscope drifts along the *x*, *y*, and *z* axes, respectively, in the body frame. ▽*_x_*, ▽*_y_*, and ▽*_z_* are accelerometer biases.

All local filters have the same state equation. The discrete-time state equation of the integrated navigation system is

(2) $$X_{k}=\Phi_{k,k-1}X_{k-1}+\Gamma_{k,k-1}W_{k-1}$$

where *k* is the discrete-time step. *X_k_* is the state vector at discrete-time *k*. Φ*_k_*_, *k*−1_ is the state transition matrix from discrete-time step *k* − 1 to *k*. Γ*_k_*_, *k*−1_ is the system noise input matrix. *W_k_*_−1_ is the process noise vector with covariance matrix *Q_k_*_−1_.

Local filter *i* is responsible for fusing subsystem *i* and INS. The discrete-time measurement equation of local filter *i* can be described by

(3) $$Z_{k}^{i}=H_{k}^{i}X_{k}+V_{k}^{i}$$

where the superscript *i* represents the state parameter of the *i*-th local filter. *Z_k_* is the measurement vector at discrete-time *k*. *H_k_* is the measurement matrix. *V_k_* is the measurement noise vector with covariance matrix *R_k_*.

### 2.3. Classical Federated Filtering Algorithm

Federated filtering includes three processes: information distribution, time update and measurement update in local filters, and information fusion in the global filter.

(1) Information distribution

The global estimation covariance matrix and the system noise covariance matrix are amplified and then fed back to the local filters.

(4) $$P_{k-1}^{i}=\beta_{i}^{-1}P_{k-1}^{g}$$

(5) $$Q_{k-1}^{i}=\beta_{i}^{-1}Q_{k-1}^{g}$$

where the superscript *g* represents the state parameter of the global filter. *β_i_* (*i* = 1, 2, …, *N*) is the information sharing coefficient, which can be determined according to a specific information distribution principle. The information sharing coefficients satisfy

(6) $$\sum_{i=1}^{N}\beta_{i}=1$$

The state estimation of local filter *i* is reset by the global state estimation.

(7) $${\hat{X}}_{k-1}^{i}={\hat{X}}_{k-1}^{g}$$

(2) Local filtering

If the local filter is implemented with a Kalman filter [23,24], the filtering process is as follows.

The time update process is

(8) $${\hat{X}}_{k,k-1}^{i}=\Phi_{k,k-1}{\hat{X}}_{k-1}^{i}$$

(9) $$P_{k,k-1}^{i}=\Phi_{k,k-1}P_{k-1}^{i}\Phi_{k,k-1}^{T}+\Gamma_{k,k-1}Q_{k-1}^{i}\Gamma_{k,k-1}^{T}$$

where ${\hat{X}}_{k,k-1}^{i}$ is the state estimation of local filter *i* after time update. $P_{k,k-1}^{i}$ is the covariance matrix of ${\hat{X}}_{k,k-1}^{i}$.

The measurement update process is

(10) $$K_{k}^{i}=P_{k,k-1}^{i}{\left(H_{k}^{i}\right)}^{T}{\left[H_{k}^{i}P_{k,k-1}^{i}{\left(H_{k}^{i}\right)}^{T}+R_{k}^{i}\right]}^{-1}$$

(11) $${\hat{X}}_{k}^{i}={\hat{X}}_{k,k-1}^{i}+K_{k}^{i}(Z_{k}^{i}-H_{k}^{i}{\hat{X}}_{k,k-1}^{i})$$

(12) $${\left(P_{k}^{i}\right)}^{-1}={\left(P_{k,k-1}^{i}\right)}^{-1}+{\left(H_{k}^{i}\right)}^{T}{\left(R_{k}^{i}\right)}^{-1}H_{k}^{i}$$

where $K_{k}^{i}$ is the gain matrix of local filter *i*. ${\hat{X}}_{k}^{i}$ is the state estimation of local filter *i* at discrete-time *k*. $P_{k}^{i}$ is the covariance matrix of ${\hat{X}}_{k}^{i}$.

(3) Information fusion
  (13) $${\left(P_{k}^{g}\right)}^{-1}=\sum_{i=1}^{N}{\left(P_{k}^{i}\right)}^{-1}$$
  (14) $${\hat{X}}_{k}^{g}=P_{k}^{g}\cdot \sum_{i=1}^{N}\left[{\left(P_{k}^{i}\right)}^{-1}{\hat{X}}_{k}^{i}\right]$$
  where $P_{k}^{g}$ is the covariance matrix of global state estimation. ${\hat{X}}_{k}^{g}$ is the global state estimation.

Finally, ${\hat{X}}_{k}^{g}$ is fed back to the reference system to correct its navigation errors, ultimately providing accurate navigation results.

## 3. Fault-Tolerant Information Fusion Algorithm

### 3.1. Fault Propagation Analysis

As previously stated, FDI modules are set up for each local filter to efficiently isolate wrong measurements and prevent the adverse effects of the faulty navigation subsystem. The FDI module developed in this paper is essentially implemented by a fault-tolerant filtering algorithm. The propagation mechanism of erroneous measurements in information fusion is analyzed first to achieve FDI.

If a subsystem malfunctioned, the faulty measurement could be represented as

(15) $${\tilde{Z}}_{k}^{i}=H_{k}^{i}X_{k}+V_{k}^{i}+f_{k}^{i}$$

where $f_{k}^{i}$ is the measurement fault at discrete-time *k*.

In the event that the measurement is inaccurate, the filter innovation is

(16) $$\begin{array}{l} {\tilde{r}}_{k}^{i}={\tilde{Z}}_{k}^{i}-H_{k}^{i}{\hat{X}}_{k,k-1}^{i}\\ \;\;\;=H_{k}^{i}X_{k}+V_{k}^{i}+f_{k}^{i}-H_{k}^{i}{\hat{X}}_{k,k-1}^{i}\\ \;\;\;=Z_{k}^{i}-H_{k}^{i}{\hat{X}}_{k,k-1}^{i}+f_{k}^{i}=r_{k}^{i}+f_{k}^{i} \end{array}$$

where $r_{k}^{i}$, which follows a zero-mean Gaussian distribution, is the filter innovation in the non-fault case.

As can be concluded from Equation (16), the measurement fault causes the change of filter innovation. The filter innovation tracks the fault $f_{k}^{i}$. If the incorrect measurement is still adopted to the measurement update of the local filter, the local state estimation contaminated by the fault is

(17) $$\begin{array}{l} {\tilde{X}}_{k}^{i}={\hat{X}}_{k,k-1}^{i}+K_{k}^{i}\cdot {\tilde{r}}_{k}^{i}\\ \;\;\;\;={\hat{X}}_{k,k-1}^{i}+K_{k}^{i}\cdot (Z_{k}^{i}-H_{k}^{i}{\hat{X}}_{k,k-1}^{i}+f_{k}^{i})\\ \;\;\;\;={\hat{X}}_{k}^{i}+K_{k}^{i}\cdot f_{k}^{i} \end{array}$$

where ${\hat{X}}_{k}^{i}$ is the local state estimation in the non-fault case.

According to Equation (17), the local state estimation will track the measurement fault $f_{k}^{i}$ by the local gain matrix $K_{k}^{i}$. If the fault is not isolated timely, the filter innovation at discrete-time *k* + 1 will be

(18) $$\begin{array}{l} {\tilde{r}}_{k+1}^{i}={\tilde{Z}}_{k+1}^{i}-H_{k+1}^{i}{\tilde{X}}_{k+1,k}^{i}\\ \;\;\;\;\;\;\;=Z_{k+1}^{i}+f_{k+1}^{i}-H_{k+1}^{i}\Phi_{k+1,k}{\tilde{X}}_{k}^{i} \end{array}$$

where $f_{k+1}^{i}$ is the measurement fault at discrete-time *k* + 1.

Substituting Equation (17) into Equation (18) yields

(19) $$\begin{array}{l} {\tilde{r}}_{k+1}^{i}={\tilde{Z}}_{k+1}^{i}-H_{k+1}^{i}{\tilde{X}}_{k+1,k}^{i}\\ \;\;\;\;\;\;\;=Z_{k+1}^{i}+f_{k+1}^{i}-H_{k+1}^{i}\Phi_{k+1,k}({\hat{X}}_{k}^{i}+K_{k}^{i}f_{k}^{i})\\ \;\;\;\;\;\;\;=Z_{k+1}^{i}-H_{k+1}^{i}{\hat{X}}_{k+1,k}^{i}+f_{k+1}^{i}-H_{k+1}^{i}\Phi_{k+1,k}K_{k}^{i}f_{k}^{i}\\ \;\;\;\;\;\;\;=r_{k+1}^{i}+f_{k+1}^{i}-H_{k+1}^{i}\Phi_{k+1,k}K_{k}^{i}f_{k}^{i} \end{array}$$

Equation (19) suggests that the fault included in the filter innovation at discrete-time *k*+1 will be decreased by $H_{k+1}^{i}\Phi_{k+1,k}K_{k}^{i}f_{k}^{i}$ if the fault is not isolated timely at discrete-time *k*. If a gradual fault occurs, the fault is small at first and is difficult to detect immediately. ${\tilde{X}}_{k+1,k}^{i}$ will track the fault $f_{k}^{i}$, making the fault in ${\tilde{r}}_{k+1}^{i}$ smaller. That is, the innovation will always be small, making it challenging to identify gradual faults. In this case, methods such as the residual chi-square detection method will fail because they detect faults based on the growth of innovation and are therefore insensitive to gradual faults [25,26]. The fault will contaminate the local state estimation following the measurement update. After the information fusion, the inaccurate global state estimation will be output. In the presence of feedback, the global state estimation may contaminate even otherwise faultless navigation subsystems. Navigation results will be seriously distorted as a result of the pollution of the entire integrated navigation system.

### 3.2. Fault-Tolerant Local Filter Based on Fuzzy Inference

According to the analysis in Section 3.1, the local state estimation will track the fault by the gain matrix. Therefore, the gain matrix is considered to be adjusted according to the reliability of the measurement so as to realize the fault tolerance filter. Then, it is necessary to find some relevant variables that reflect the quality of measurements.

Although the residual chi-square test is not sensitive to slowly changing gradual faults, it is sensitive to abrupt faults. The detection statistic of the residual chi-square test is

(20) $$\lambda_{k}^{i}={\left(r_{k}^{i}\right)}^{T}{\left(P_{r,k}^{i}\right)}^{-1}r_{k}^{i}$$

where $r_{k}^{i}$ is the filter innovation. $P_{r,k}^{i}$ is the covariance matrix of $r_{k}^{i}$. Concretely,

(21) $$\;r_{k}^{i}=Z_{k}^{i}-H_{k}^{i}{\hat{X}}_{k,k-1}^{i}$$

(22) $$P_{r,k}^{i}=H_{k}^{i}P_{k,k-1}^{i}{\left(H_{k}^{i}\right)}^{T}+R_{k}^{i}$$

$\lambda_{k}^{i}$ follows the chi-square distribution when there is no fault. When a fault occurs, $r_{k}^{i}$ is no longer a zero-mean white noise process, then $\lambda_{k}^{i}$ will be larger. Consequently, $\lambda_{k}^{i}$ can be compared with the predetermined threshold $T_{d}^{i}$. Both the missed and false detection rates are used to set the threshold. A new parameter $\alpha_{k}^{i}$ is defined in light of this.

(23) $$\alpha_{k}^{i}=\frac{\lambda_{k}^{i}}{T_{d}^{i}}$$

There must be a fault if $\alpha_{k}^{i}$ is significantly higher than 1. The likelihood of failure is greatly increased if $\alpha_{k}^{i}$ is near 1. There is little chance of an abrupt fault if $\alpha_{k}^{i}$ is much smaller than 1. It is worth noting that the fault tracking outlined in Section 3.1 may cause $\alpha_{k}^{i}$ to remain much less than 1 even in the case of a gradual fault. Therefore, only using the parameter $\alpha_{k}^{i}$ as the basis for judging whether there is a fault is not reliable. It is necessary to further explore a parameter that is sensitive to gradual faults.

Since $P_{k,k-1}^{i}$ is the outcome of the time update process, the theoretical covariance matrix of innovation is $P_{r,k}^{i}$, which is calculated by Equation (22). On the other hand, using the innovation sequence in the moving window yields the maximum likelihood estimation of the actual covariance matrix.

(24) $${\hat{P}}_{r,k}^{i}=\frac{1}{M}\sum_{j=0}^{M-1}r_{k-j}^{i}{\left(r_{k-j}^{i}\right)}^{T}$$

where ${\hat{P}}_{r,k}^{i}$ is the actual covariance matrix of innovation. *M* is the length of the moving window.

$P_{r,k}^{i}$ will not be affected by the fault if there is no fault at discrete-time *k* − 1 and a gradual fault occurs at discrete-time *k*. However, ${\hat{P}}_{r,k}^{i}$ will immediately hold the fault information. ${\hat{P}}_{r,k}^{i}$ will therefore deviate from $P_{r,k}^{i}$. As time goes on, the innovations in the moving window will include an increasing amount of defect information, and the deviation degree of ${\hat{P}}_{r,k}^{i}$ from $P_{r,k}^{i}$ will get increasingly severe. This will facilitate the detection of gradual faults. The likelihood of failure increases with the degree of deviation. Thus, the reliability of the measurements can be assessed by calculating the degree of deviation between the theoretical and the actual covariance matrices of innovation. This leads to the definition of a parameter $\eta_{k}^{i}$.

(25) $$\eta_{k}^{i}=\frac{{\left\|{\hat{P}}_{r,k}^{i}\right\|}_{F}}{{\left\|P_{r,k}^{i}\right\|}_{F}}$$

where ${\|\;\|}_{F}$ is the Frobenius norm calculation operator. Theoretically, the system performs well if $\eta_{k}^{i}$ is close to 1. The likelihood of failure increases with the degree of $\eta_{k}^{i}$ deviation from 1. However, in fact, the actual covariance and the theoretical covariance of the innovation cannot be exactly consistent. Therefore, if $\eta_{k}^{i}$ is in a certain interval not far greater than 1, it can be considered that the system works well.

In summary, both parameters $\alpha_{k}^{i}$ and $\eta_{k}^{i}$ can identify whether the navigation subsystem is faulty. A new variable that measures the reliability of the measurements is defined as the observational quality factor $\omega_{k}^{i}$. The filtering gain matrix can be adjusted using the observational quality factor to isolate the faults. Then, it is necessary to establish the quantitative relationship between the observational quality factor $\omega_{k}^{i}$ and the fault determination-related variables $\alpha_{k}^{i}$ and $\eta_{k}^{i}$. The evaluation of $\alpha_{k}^{i}$ and $\eta_{k}^{i}$’s degree of divergence from 1 is vague, just like in the earlier analysis. In addition, the term “reliability of measurement” is also vague. The membership relationship of the observational quality factor to the working state of the subsystem is flexible. Rather than being evaluated as either valid or invalid, the measurement is assessed by its extent of validity. In order to establish the relationship between these three variables, a fuzzy inference system [27,28] is appropriate in this case. Specifically,

(26) $$\omega_{k}^{i}=f(\alpha_{k}^{i},\eta_{k}^{i})$$

where *f*( , ) is the fuzzy inference function.

**Figure 2:**
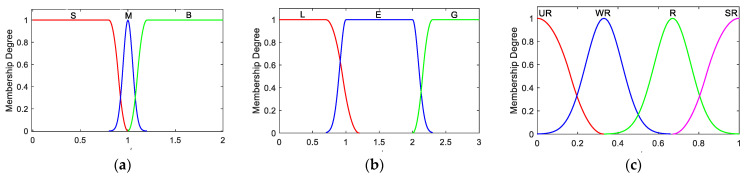
Membership functions. (**a**) $\alpha_{k}^{i}$; (**b**) $\eta_{k}^{i}$; (**c**) $\omega_{k}^{i}$.

The fuzzy inference system corresponding to the local filter *i* is designed with two inputs and one output. One of the inputs, $\alpha_{k}^{i}$, is described by the linguistic variables Small (S), Medium (M), and Big (B). The linguistic variables Less (L), Equal (E), and Greater (G) describe the other input $\eta_{k}^{i}$. The output $\omega_{k}^{i}$, which measures the reliability of the measurements, is characterized by the linguistic variables Unreliable (UR), Weak Reliable (WR), Reliable (R), and Strong Reliable (SR). Actually, the four linguistic variables of $\omega_{k}^{i}$ correspond to four working states of the navigation subsystem, namely, major fault, slight fault, possible fault, and no fault. Figure 2 shows the fuzzy membership functions of $\alpha_{k}^{i}$, $\eta_{k}^{i}$, and $\omega_{k}^{i}$ respectively. The membership functions of $\alpha_{k}^{i}$ and $\eta_{k}^{i}$ are determined based on the relationship between the values of $\alpha_{k}^{i}$ and $\eta_{k}^{i}$ and the fault probability as described above. The Gaussian membership function, which has good continuity and smoothness, is selected as the membership function of $\omega_{k}^{i}$.

If $\alpha_{k}^{i}$ is Big, then the measurements are unreliable since an abrupt fault has occurred, regardless of $\eta_{k}^{i}$. There may be a gradual fault present if $\alpha_{k}^{i}$ is Medium and $\eta_{k}^{i}$ is Less or Greater, in which case the measurements are weakly reliable. If $\alpha_{k}^{i}$ is Small and $\eta_{k}^{i}$ is Equal, the subsystem works well, and then the measurements are strongly reliable. Fuzzy inference rules can be derived by analogy. Table 1 provides the fuzzy rule base.

Based on the fuzzy rules in Table 1, the relationship between $\alpha_{k}^{i}$, $\eta_{k}^{i}$, and the observational quality factor $\omega_{k}^{i}$ can be determined using the fuzzy reasoning process. First, make the inputs $\alpha_{k}^{i}$ and $\eta_{k}^{i}$ fuzzy according to the membership function in Figure 2a and b, respectively. The linguistic variables to which they belong and the corresponding membership degree are identified. The Mamdani max–min inference rule [29] then determines the linguistic variables to which $\omega_{k}^{i}$ belongs and the corresponding membership degree based on the fuzzy inference rules in Table 1. Lastly, the centroid method is applied to defuzzify the results based on the membership function shown in Figure 2c. The quantification result of the observational quality factor $\omega_{k}^{i}$ can be computed finally.

As the observational quality factor can measure the reliability of the measurements, it is adopted to adjust the filtering gain matrix to isolate the inaccurate measurements.

(27) $${\tilde{K}}_{k}^{i}=\omega_{k}^{i}P_{k,k-1}^{i}{\left(H_{k}^{i}\right)}^{T}{\left[H_{k}^{i}P_{k,k-1}^{i}{\left(H_{k}^{i}\right)}^{T}+R_{k}^{i}\right]}^{-1}$$

where $\omega_{k}^{i}\in [0,1]$. Equation (10) in the measurement update process is substituted by Equation (27). In other words, the filter gain matrix is modified online according to the reliability of the measurements. The filter gain matrix is a classical representation when $\omega_{k}^{i}$ equals 1. According to fuzzy inference, the value of $\omega_{k}^{i}$ decreases as the quality of measurement information declines. The filter gain matrix will thereafter drop as well. It is evident that the utilization of measurements by the local state estimation will decrease at this point when combined with Equation (11). It relies more on one-step state estimations for updates. This isolates the faulty subsystem by masking inaccurate measurements. In conclusion, the FDI module is implemented by fuzzy inference, and local filtering is enhanced to ensure fault tolerance.

### 3.3. Adaptive Information Sharing

In order to increase the accuracy of multi-source information fusion, a federated filter works in the feedback mode. However, there is a risk that the feedback from the global filter to the local filters will cross-infect the fault. Therefore, in order to design a reasonable information distribution strategy, it is necessary to assess the influence of information sharing coefficients on the fault detection ability and fault tolerance performance of the system.

Substituting Equations (7) and (8) into Equation (11), the state estimation of local filter *i* is expressed as follows.

(28) $${\hat{X}}_{k}^{i}=(I-K_{k}^{i}H_{k}^{i})\cdot \Phi_{k,k-1}\cdot {\hat{X}}_{k-1}^{g}+K_{k}^{i}\cdot Z_{k}^{i}$$

In addition to Equation (12), the covariance matrix of ${\hat{X}}_{k}^{i}$ can also be expressed as

(29) $$P_{k}^{i}=(I-K_{k}^{i}H_{k}^{i})\cdot P_{k,k-1}^{i}$$

In addition,

(30) $$P_{k,k-1}^{i}=\beta_{i}^{-1}\cdot P_{k,k-1}^{g}$$

where $P_{k,k-1}^{g}$ is the covariance matrix of the global one step estimation.

We substitute Equations (12), (29) and (30) and the improved gain matrix Equation (27) into Equation (28).

(31) $$\begin{array}{l} {\hat{X}}_{k}^{i}=[{{(P}_{k,k-1}^{g)}}^{-1}+\beta_{i}^{-1}{\left(H_{k}^{i}\right)}^{T}{\left(R_{k}^{i}\right)}^{-1}H_{k}^{i}]-1{\left(P_{k,k-1}^{g}\right)}^{-1}\Phi_{k,k-1}\cdot {\hat{X}}_{k-1}^{g}\\ \;\;\;\;\;+\omega_{k}^{i}[{\left(H_{k}^{i}\right)}^{-1}+\beta_{i}^{-1}P_{k,k-1}^{g}{\left(H_{k}^{i}\right)}^{T}{\left(R_{k}^{i}\right)}^{-1}]\cdot Z_{k}^{i}\\ \;\;\;\;\;=\eta_{x}\cdot {\hat{X}}_{k-1}^{g}+\eta_{z}\cdot Z_{k}^{i} \end{array}$$

where *η_z_* and *η_x_* represent the utilization of the local state estimation to its own local measurement information and to the global state estimation, respectively. Equation (31) states that *η_x_* is proportional to *β_i_*. *η_z_* is proportional to $\omega_{k}^{i}$, but inversely proportional to *β_i_*.

If the navigation subsystem *i* fails at discrete-time *k*, the dependence of the local state estimate ${\hat{X}}_{k}^{i}$ on the global state estimate ${\hat{X}}_{k-1}^{g}$ should increase, and the utilization of measurement information $Z_{k}^{i}$ should decrease. In other words, *η_x_* should be larger and *η_z_* should be smaller. According to the improved gain matrix designed in Section 3.2, $\omega_{k}^{i}$ will decrease when the fault occurs, followed by a fall in *η_z_*, which is in accordance with the fault isolation requirements. Then, for *β_i_*, it needs to be designed to increase in case of failure. As *β_i_* increases, *η_x_* increases, and *η_z_* decreases, the fault-tolerant logic requirements are satisfied. Consequently, the information sharing coefficient *β_i_* is defined as

(32) $$\beta_{i}=\frac{\sum_{j=1}^{N}\omega_{k}^{j}-\omega_{k}^{i}}{(N-1)\sum_{j=1}^{N}\omega_{k}^{j}}$$

where *N* is the total number of local filters, that is, the number of auxiliary navigation subsystems. The definition of *β_i_* satisfies Equation (6). If subsystem *i* fails, $\omega_{k}^{i}$ will be smaller, then *β_i_* will be larger.

The influence of the adaptive information sharing coefficients on fault detection needs to be analyzed. According to the previous definition, if a subsystem *i* fails, *β_i_* will increase. It follows from Equation (30) that $P_{k,k-1}^{i}$ will decrease. $P_{r,k}^{i}$ will decrease in accordance with Equation (22). $\lambda_{k}^{i}$ will also increase based on Equation (20). Then, according to Equation (23), $\alpha_{k}^{i}$ will increase, which facilitates fault detection. Furthermore, based on Equation (25), the denominator of $\eta_{k}^{i}$ will be reduced, which would enhance sensitivity to innovation changes and make it easier to identify gradual faults. The rationality and advantages of the adaptive information distribution strategy defined in Equation (32) are confirmed by the analysis.

### 3.4. Fault-Tolerant Fltering Algorithm

**Figure 3:**
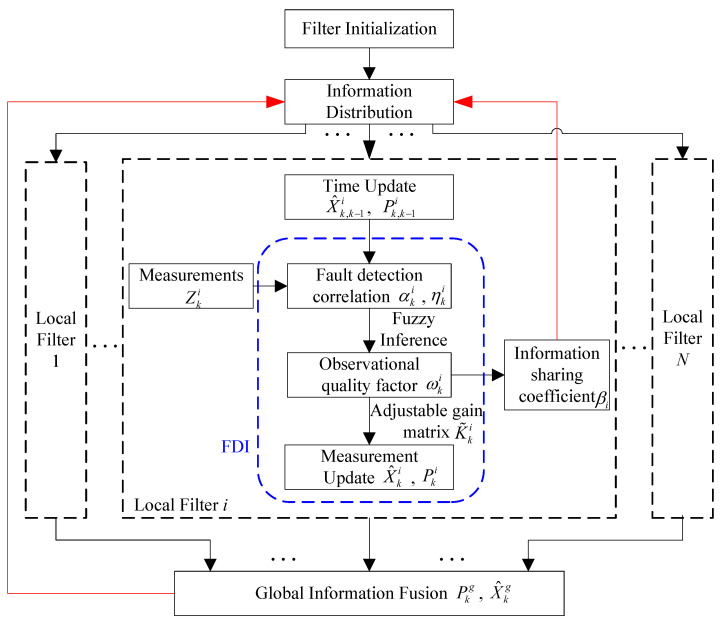
Flow chart of fault-tolerant information fusion algorithm.

Figure 3 displays the fault-tolerant information fusion algorithm flow based on the feedback federated filter, which relies on the design in Section 3.2 and Section 3.3. In order to improve the estimation accuracy of the local filter, the global estimation of the previous time is utilized for information distribution. The time update of the local filter is carried out in accordance with the traditional Kalman filter. The FDI module is configured to quickly detect and isolate both abrupt and gradual faults. Since incorrect measurement will impact the filtering innovation, two fault detection correlation quantities $\alpha_{k}^{i}$ and $\eta_{k}^{i}$ are constructed. They are applied as inputs to a fuzzy inference system. The observation quality factor $\omega_{k}^{i}$ output by the fuzzy system can measure the reliability of the measurements. The filter gain matrix is improved by $\omega_{k}^{i}$, so that it can adjust the utilization rate of the measurements in real time. Finally, the measurement update process yields the local state estimation. Essentially, the fuzzy inference system and the adjustable gain matrix, respectively, are responsible for fault detection and isolation. In addition, observation quality factors $\omega_{k}^{j}\;\;(j=1,\cdots N)$ determine the information sharing coefficient *β_i_*, which can guarantee the dependability of fault isolation and further improve the sensitivity of fault detection. The global state estimation ${\hat{X}}_{k}^{g}$ is finally obtained by fusing the reliable local state estimations using the global filter.

## 4. Simulation Verification and Result Analysis

**Figure 4:**
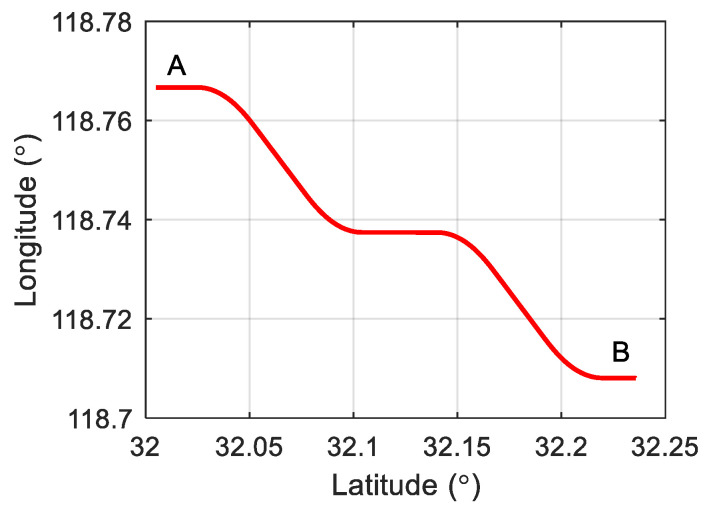
Simulated trajectory.

**Figure 5:**
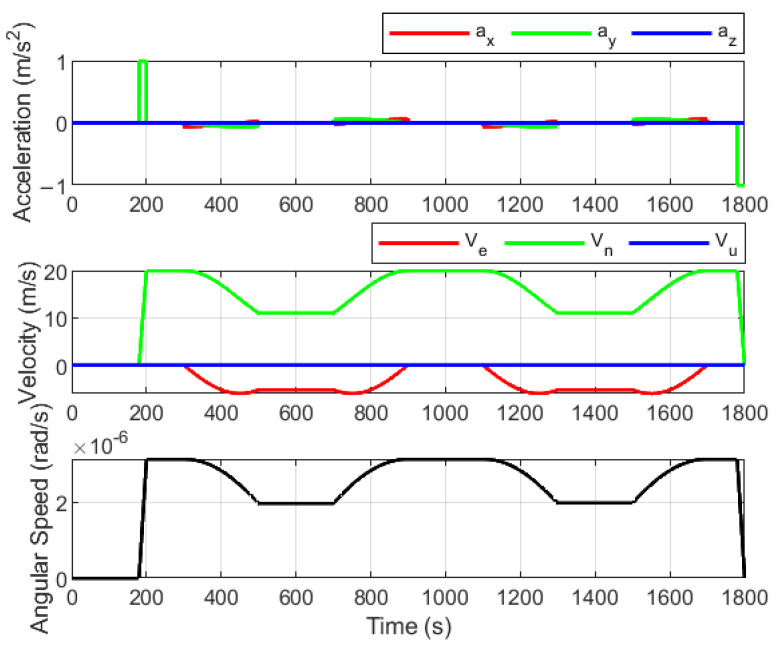
Acceleration profile, velocity profile, and angular speed profile.

To evaluate the performance of the proposed fault-tolerant information fusion method, simulation experiments were conducted. The proposed algorithm was applied to the SINS/OD/GPS integrated navigation system of an autonomous vehicle. The simulated trajectory is depicted in Figure 4. The total length of the trajectory is 26,662.6 m over 1800 s. The initial position of the test vehicle is at a latitude of 32°03′ and a longitude of 118°46′. The vehicle travels from point A to point B, performing a series of maneuvers, including acceleration, deceleration, left turns, and right turns. The acceleration profile, velocity profile, and angular speed profile are shown in Figure 5. The gyroscope bias error and random walk error are set to 0.03°/h and 0.005°/$\sqrt{h}$, respectively. The accelerometer bias error and random walk error are set to 0.2 mg and 50 μg/$\sqrt{\mathrm{Hz}}$, respectively. The position measurement error of the GPS is assumed to be 2 m, while the velocity measurement error of the OD is set at 0.1 m/s. The SINS solution period is 10 ms, and the filter measurement update period is 1 s.

**Figure 6:**
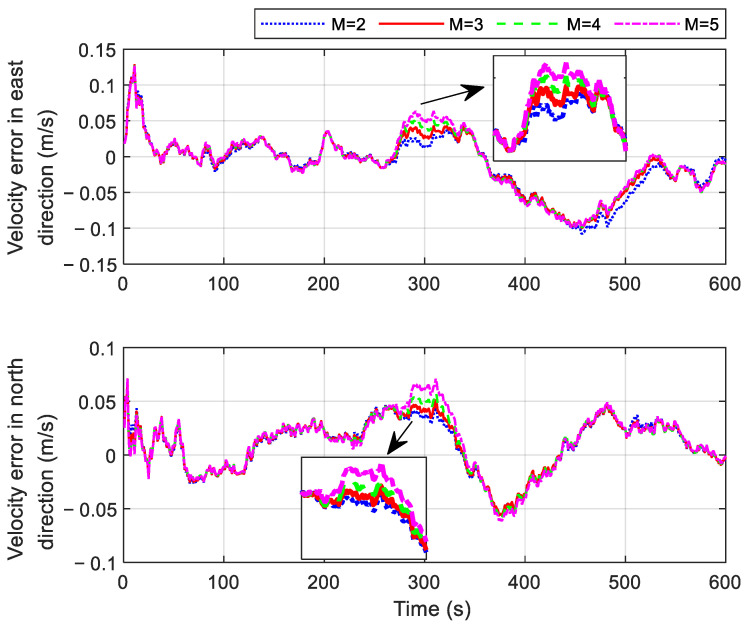
Velocity errors in cases with different *M* values.

**Figure 7:**
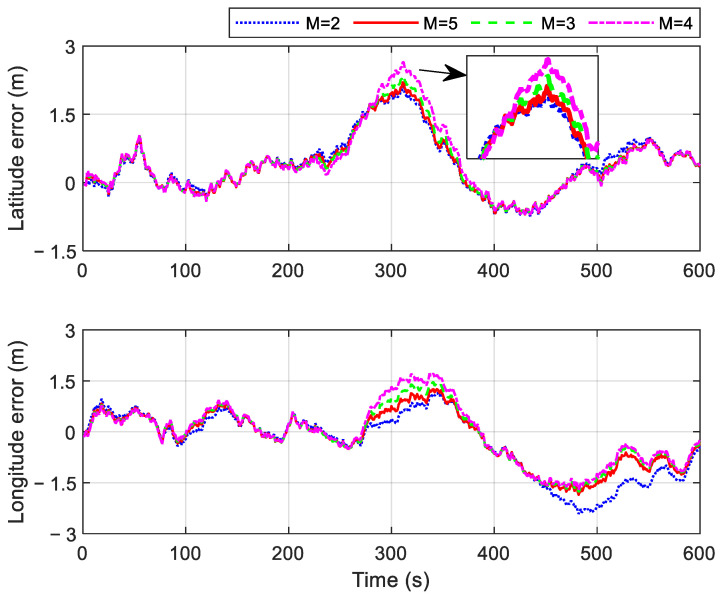
Position errors in cases with different *M* values.

The actual covariance matrix ${\hat{P}}_{r,k}^{i}$ is related to the length of the moving window *M*, as shown in Equation (24). During the initial stage of gradual fault occurrence, the fault information $f_{k}^{i}$ contained in the innovation ${\tilde{r}}_{k}^{i}$ is small. If the *M* value is too small, the fault judgment may be influenced by random noise in the data. On the other hand, setting an excessively high *M* value results in two drawbacks: first, it significantly increases the computational load, and second, it may lead to an increased fault detection delay. Therefore, the selection of the *M* value must balance detection sensitivity and computational efficiency. In this study, the optimal *M* value is determined through a series of simulation experiments. To simulate a gradual fault, an error with a 0.0008 m/s^2^ change rate is introduced into the OD outputs between 250 s and 300 s. The resulting velocity errors and the position errors for different *M* values are presented in Figure 6 and Figure 7, respectively.

As shown in Figure 6 and Figure 7, the velocity and position errors gradually increase with larger values of *M* during the set fault period. This occurs because a larger *M* leads to longer fault detection delays, allowing faulty OD measurements to have a greater impact on navigation results. Interestingly, both velocity and position errors also increase during certain non-fault periods when *M* = 2. For example, the east velocity error between 450 s and 550 s, the latitude error between 500 s and 550 s, and the longitude error between 450 s and 580 s all rise significantly. This is due to the small value of *M*, which makes the system more susceptible to random noise overwhelming the valid statistics of the innovation. This misjudgment of fault status ultimately reduces the accuracy of navigation results. To balance the timeliness of gradual fault detection and computational efficiency, *M* is set to 3 in the subsequent simulation.

To evaluate the performance of the proposed fault-tolerant information fusion method, several typical faults were introduced into the integrated navigation system. Table 2 provides the parameters used for fault simulation. Zero outputs were used to simulate a complete subsystem failure, such as one caused by a power outage. Another fault scenario involved GPS output freezing just before signal loss, which occurs when satellite signals are blocked, such as when a vehicle passes through a tunnel. This scenario was simulated between 750 s and 770 s. Gradual faults were also considered, such as those caused by GPS pseudo-range increases due to clock drift. These fault scenarios were specifically designed to test the robustness and effectiveness of the proposed method under different conditions.

**Figure 8:**
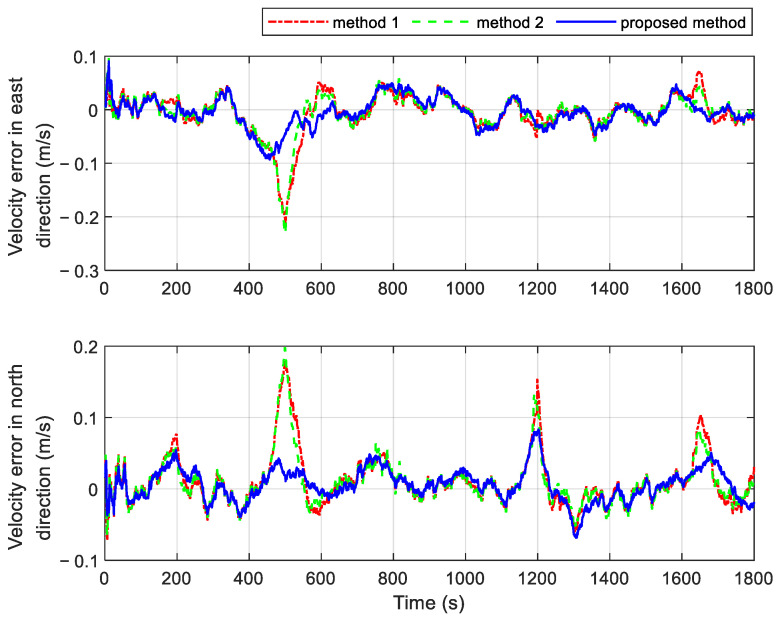
Velocity errors of different methods in cases with multiple faults.

**Figure 9:**
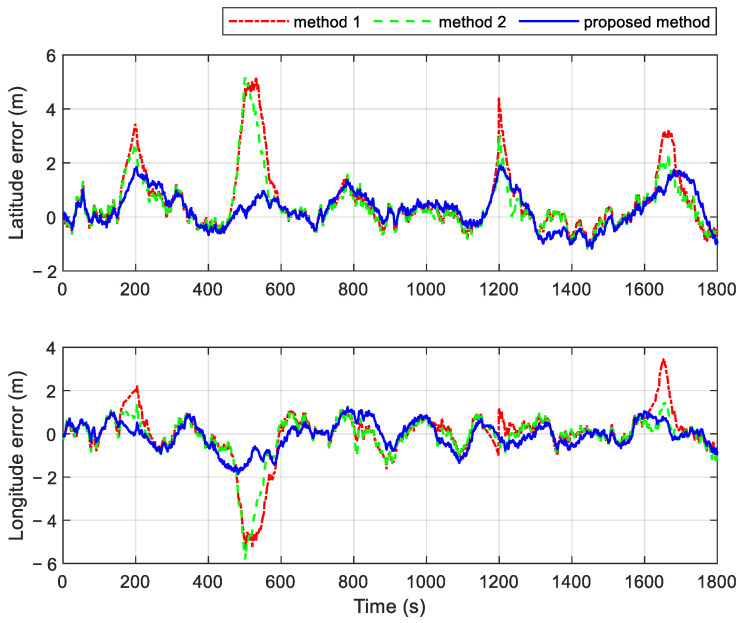
Position errors of different methods in cases with multiple faults.

Two additional fault-tolerant methods are introduced for comparison with the proposed method. Both comparison methods utilize the residual chi-square test for fault detection. In Method 1, the faulty local branch is immediately isolated upon fault detection, meaning that the integrated navigation relies solely on the fault-free subsystem. In contrast, Method 2 incorporates one-step prediction state estimation and its covariance into the information fusion process after a fault is identified. It is important to note that both methods 1 and 2 operate within a federated filter framework without feedback. This design helps prevent the rapid divergence of navigation results during fault periods. The velocity and position errors of the different methods are shown in Figure 8 and Figure 9, respectively. Table 3 provides the statistics of navigation errors obtained using three different methods under the fault conditions set in Table 2. As seen from the results, the proposed method shows significant superiority. Additionally, Table 4 provides a statistical summary of navigation errors during various GPS fault periods.

The system employing the proposed method exhibits smaller velocity and position errors during the gradual fault periods of 150 s~200 s and 450 s~500 s, as shown in Figure 8 and Figure 9. This improvement is attributed to the proposed method’s higher sensitivity to gradual faults. It can quickly and efficiently isolate gradual issues by adjusting the observational quality factors, which are derived from fuzzy inference based on the subsystem’s operational state. In contrast, the residual chi-square test requires more time to identify gradual faults. During this delay, inaccurate measurement information is used in the measurement update of the local filter, which subsequently impacts the global fusion result through information fusion. As a result, the navigation errors of systems using Methods 1 and 2 are significantly higher.

The navigation results of the three methods are similar during the periods of 750 s~770 s and 1010 s~1030 s, with the velocity and position errors not increasing significantly. This is because both the residual chi-square test and the fault detection based on fuzzy inference are more sensitive to abrupt faults. The fault isolation process improves the navigation results by promptly detecting the abrupt faults. Once the abrupt fault is detected in time, better navigation results can be achieved through fault isolation.

The duration of abrupt faults increases during the intervals of 1160 s~1200 s and 1600 s~1650 s. Different fault isolation strategies yield varied navigation outcomes, despite all three methods being capable of detecting abrupt faults rapidly. Method 1, which relies solely on another non-faulty subsystem to assist the INS during fault occurrences, produces the largest navigation errors. As the fault duration increases, the accuracy of the navigation results deteriorates significantly. In contrast, Method 2 employs one-step state estimation and incorporates its covariance after the time update into the information fusion process. This approach mitigates the rapid divergence of navigation results, leading to more accurate outcomes compared to Method 1. Notably, the proposed method achieves the smallest navigation errors during extended periods of abrupt failure. It isolates faults by dynamically adjusting the gain matrix of the local filter in real time and adaptively tuning the feedback federated filtering via the information sharing coefficients. This adaptive strategy significantly enhances the accuracy of the information fusion results.

**Figure 10:**
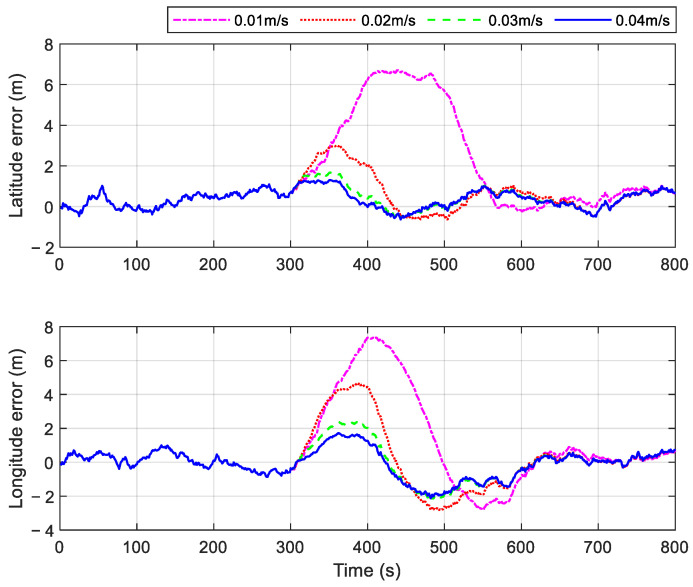
Position errors of the proposed method in cases with different gradual faults.

**Figure 11:**
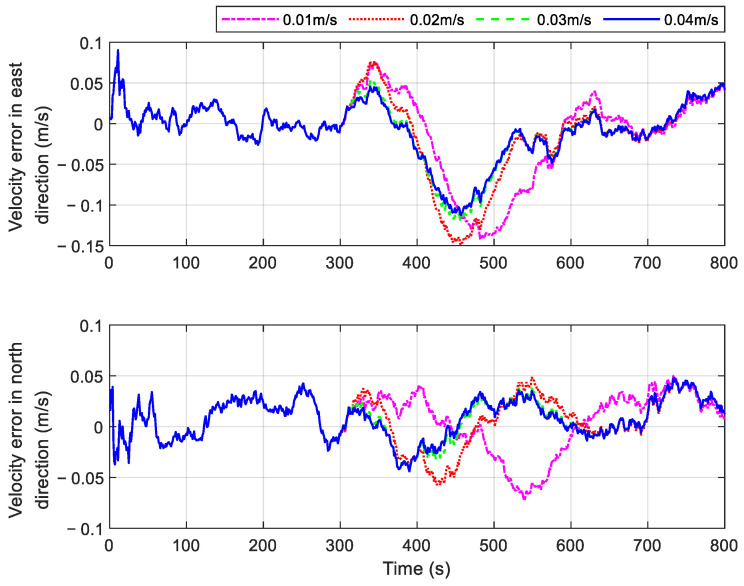
Velocity errors of the proposed method in cases with different gradual faults.

A gradual fault can be numerically represented as a ramp function superimposed on the measured data. When the slope of the ramp function is smaller, the incremental effect of the gradual fault becomes less apparent, posing challenges for the rapid detection of such faults. To evaluate the effectiveness of the proposed method, several gradual faults with different growth rates were simulated. Specifically, gradual faults with change rates of 0.01 m/s, 0.02 m/s, 0.03 m/s, and 0.04 m/s were introduced into the GPS outputs from 300 s to 400 s. The position errors and velocity errors of the proposed method under these conditions are illustrated in Figure 10 and Figure 11. The simulation results indicate that navigation errors increase as the growth rate of the gradual fault decreases. This occurs because smaller gradual fault values result in lower fault-related information within the filter innovation, making detection more challenging. However, as shown in Figure 10 and Figure 11, the navigation errors of the system using the proposed method remain within an acceptable range when the gradual fault change rate added to the GPS output is 0.03 m/s or higher.

**Figure 12:**
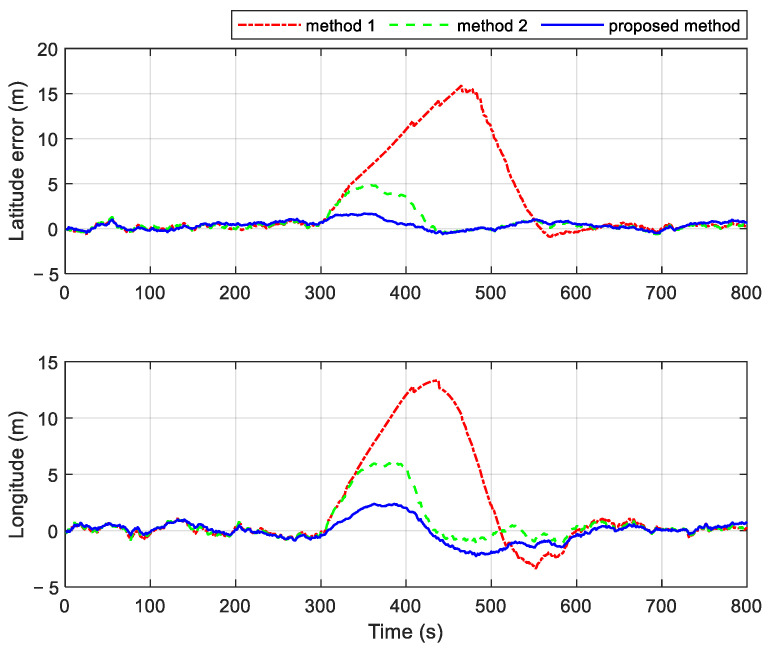
Position errors of different methods in cases with a gradual fault.

**Figure 13:**
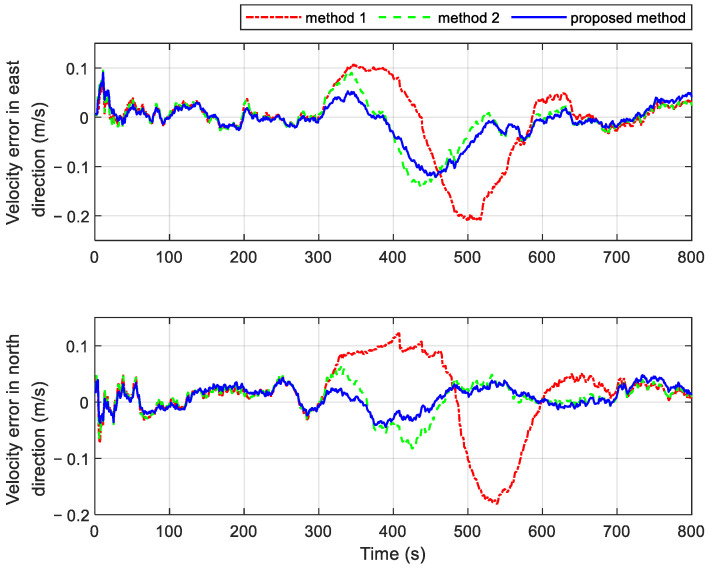
Velocity errors of different methods in cases with a gradual fault.

Based on the above experiments, a GPS gradual fault with a 0.03 m/s change rate was introduced to test three different methods. Figure 12 and Figure 13 show the resulting position and velocity errors, respectively. A gradual fault with a 0.03 m/s rate of change represents a minor fault. In this extreme case, the residual chi-square fault detection method fails to detect the fault due to the fault-following phenomenon during the detection process. As a result, the position navigation errors of Methods 1 and 2 increase rapidly after the fault occurs. Method 2, which fuses GPS branch information using the filtering time update results, shows a slower growth rate in position error compared to Method 1. Since the OD remains functional, the differences in velocity errors among the methods are less pronounced than those in position errors. However, the results clearly demonstrate the advantages of the proposed method, as it effectively mitigates the impact of the gradual fault, maintaining better navigation accuracy compared to the other methods.

Based on simulation experiments and result analysis, the proposed information fusion method significantly improves the fault tolerance and reliability of integrated navigation systems.

## 5. Conclusions

To enhance the fault-tolerant capability of integrated navigation systems, this paper proposes a novel information fusion method. The method fuses multiple navigation sensors or subsystems using a federated filter with feedback. A fault detection and isolation (FDI) module is established for each local filter, with fault detection implemented via fuzzy inference. Fault detection is achieved through fuzzy inference, which establishes a relationship between the filter innovation correlation and the observation quality factor. For fault isolation, the gain matrix of the local filter is dynamically adjusted in real time based on observation quality. Additionally, the observation quality factor determines the information sharing coefficient, ensuring dependable fault isolation while further enhancing the sensitivity of fault detection, particularly for gradual faults. A series of typical simulations were conducted to evaluate the performance of the proposed method. The results demonstrate that the method effectively addresses both abrupt and gradual faults, significantly improving the reliability of the integrated navigation system.

The limitation of this paper is that the proposed method has been primarily validated through simulations with a relatively limited trajectory distance. Therefore, the generalizability of the results to large-scale, real-world environments still needs further validation. Future work will involve testing the proposed method in more complex real-world environments to assess its robustness under varying conditions such as different weather, terrain, and sensor reliability. In terms of applications, the proposed method is most suitable for autonomous navigation systems, including autonomous vehicles, UAVs, and underwater robots, where fault tolerance and accurate state estimation are crucial. This method is particularly useful in environments with high uncertainty and sensor limitations, such as urban navigation or remote areas where GPS signals are weak or unreliable. It can also be applied in fields requiring high safety standards, such as aviation or maritime navigation, where it is essential to ensure the system remains reliable even in the presence of sensor faults.

## Figures and Tables

**Table 1:** Fuzzy inference rules.

$\omega_{k}^{i}$		$\alpha_{k}^{i}$		
		S	M	B
$\eta_{k}^{i}$	L	R	WR	UR
	E	SR	R	UR
	G	R	WR	UR

**Table 2:** Fault parameters.

Time	Faulty Subsystem	Fault Type	Fault Description
150 s~200 s	GPS	Gradual	An error with a 0.06 m/s change rate is added to the GPS outputs.
450 s~500 s	OD	Gradual	An error with a 0.0008 m/s^2^ change rate is added to the OD outputs.
75 0s~770 s	GPS	Abrupt	The GPS output is frozen at its 749 s value.
1010 s~1030 s	OD	Abrupt	The OD switches to zero outputs.
1160 s~1200 s	GPS	Abrupt	A 50 m constant error is added to the GPS outputs.
1600 s~1650 s	OD	Abrupt	A 1 m/s constant error is added to the OD outputs.

**Table 3:** Statistics of navigation errors obtained using three different methods under the fault conditions set in Table 2.

Parameter		Method 1	Method 2	Proposed Method
*δV_e_* (m/s)	Max	−0.213	−0.231	−0.093
	STD	0.038	0.036	0.027
*δV_n_* (m/s)	Max	0.178	0.198	0.083
	STD	0.037	0.034	0.023
*δL* (m)	Max	5.182	5.145	1.908
	STD	1.153	0.970	0.652
*Δλ* (m)	Max	−5.332	−5.813	−1.861
	STD	1.189	0.974	0.620

**Table 4:** Statistics of navigation errors at GPS fault stages. (*δV_e_* and *δV_n_* are velocity errors in the east and north directions, respectively. *δL* and *δλ* are latitude error and longitude error, respectively).

Parameter		150~200 s		750~770 s		1160~1200 s	
		Max	STD	Max	STD	Max	STD
Method 1	*δV_e_* (m/s)	0.025	0.0090	0.051	0.0088	−0.052	0.0120
	*δV_n_* (m/s)	0.077	0.0180	0.048	0.0151	0.154	0.0340
	*δL* (m)	3.443	1.0070	1.273	0.2714	4.446	1.0100
	*Δλ* (m)	2.068	0.6201	1.132	0.2827	1.182	0.4653
Method 2	*δV_e_* (m/s)	0.022	0.0086	0.054	0.0076	−0.038	0.0198
	*δV_n_* (m/s)	0.060	0.0121	0.066	0.0145	0.132	0.0321
	*δL* (m)	2.729	0.8146	0.979	0.1203	2.850	0.7238
	*Δλ* (m)	1.081	0.3418	1.157	0.2923	0.392	0.2408
Proposed Method	*δV_e_* (m/s)	0.021	0.0085	0.047	0.0046	−0.032	0.0110
	*δV_n_* (m/s)	0.054	0.0087	0.047	0.0057	0.080	0.0207
	*δL* (m)	1.774	0.4836	0.961	0.1181	1.787	0.5354
	*Δλ* (m)	0.589	0.2039	0.955	0.2192	0.384	0.1787

## Data Availability

The data presented in this study are available on request from the corresponding author.

## References

[1] Kartal S.K., Leblebicioglu M.K., Ege E. (2019). Experimental test of vision-based navigation and system identification of an unmanned underwater survey vehicle (SAGA) for the yaw motion. Trans. Inst. Meas. Control.

[2] Gryte K., Bryne T.H., Johansen T.A. (2021). Unmanned aircraft flight control aided by phased-array radio navigation. J. Field Robot..

[3] Savkin A.V., Verma S.C., Anstee S. (2022). Optimal navigation of an unmanned surface vehicle and an autonomous underwater vehicle collaborating for reliable acoustic communication with collision avoidance. Drones.

[4] Indelman V., Williams S., Kaess M., Dellaert F. (2013). Information fusion in navigation system via factor graph based incremental smoothing. Robot. Auton. Syst..

[5] Qin T., Li P.L., Shen S.J. (2018). VINS-Mono: A robust versatile monocular visual-inertial state estimator. IEEE Trans. Robot..

[6] Shamwell E.J., Lindgren K., Leung S., Nothwang W.D. (2020). Unsupervised deep visual-inertial odometry with online error correction for RGB-D imagery. IEEE Trans. Pattern Anal..

[7] Xu Y.F., Wang Y., Huang R. (2022). Unsupervised learning of depth estimation and camera pose with multi-scale GANs. IEEE Trans. Intell. Transp..

[8] Adusumilli S., Bhatt D., Wang H. (2013). A low-cost INS/GPS integration methodology based on random forest regression. Expert Syst. Appl..

[9] Zhao L., Kang Y.Y., Cheng J.H., Wu M.Y. (2019). A fault-tolerant polar grid SINS/DVL/USBL integrated navigation algorithm based on the centralized filter and relative position measurement. Sensors.

[10] Carlson N.A. Federated filter for fault-tolerance navigation systems. Proceedings of the IEEE PLANS ’88, Position Location and Navigation Symposium, Record. ‘Navigation into the 21st Century’.

[11] Liu Y.T., Xu X.S., Liu X.X. (2016). A fast gradual fault detection method for underwater integrated navigation systems. J. Navig..

[12] Sun R., Cheng Q., Wang G.Y. (2017). A novel online data-driven algorithm for detecting UAV navigation sensor faults. Sensors.

[13] Yang B., Liu F., Xue L., Shan B. (2023). Fault-tolerant SINS/Doppler Radar/Odometer integrated navigation method based on two-stage fault detection structure. Entropy.

[14] Park S.G., Jeong H.C., Kim J.W. (2011). Magnetic compass fault detection method for GPS/INS/Magnetic compass integrated navigation systems. Int. J. Control Autom..

[15] Williamson W.R., Speyer J.L., Sharp J. (2009). Fault detection and isolation for deep space satellites. J. Guid. Control Dynam..

[16] Ahn J., Rosihan R., Won D.H. (2011). GPS integrity monitoring method using auxiliary nonlinear filters with log likelihood ratio test approach. J. Electr. Eng. Technol..

[17] Gao G.L., Gao S.S., Hu G.G., Peng X. (2021). Spectral redshift observation-based SINS/SRS/CNS integration with an adaptive fault-tolerant cubature Kalman filter. Meas. Sci. Technol..

[18] Jiang W., Li Y., Rizos C. (2017). A multisensor navigation system based on an adaptive fault-tolerant GOF algorithm. IEEE Trans. Intell. Transp..

[19] Liang Y.Q., Jia Y.M. (2015). A nonlinear quaternion-based fault-tolerant SINS/GNSS integrated navigation method for autonomous UAVs. Aerosp. Sci. Technol..

[20] Kang J., Xiong Z., Wang R., Zhang L. (2022). Multi-layer fault-tolerant robust filter for integrated navigation in launch inertial coordinate system. Aerospace.

[21] Mohammadi A., Sheikholeslam F., Emami M., Mirjalili S. (2023). Designing INS/GNSS integrated navigation systems by using IPO algorithms. Neural Comput. Appl..

[22] Zhu J.P., Li A., Qin F.J. (2022). A hybrid method for dealing with DVL faults of SINS/DVL integrated navigation system. IEEE Sens. J..

[23] Chen K., Zhang P.T., You L., Sun J. (2024). Research on Kalman filter fusion navigation algorithm assisted by CNN-LSTM neural network. Appl. Sci..

[24] Or B., Klein I. (2022). A hybrid model and learning-based adaptive navigation filter. IEEE Trans. Instrum. Meas..

[25] Zhao G.L., Wang J.B., Gao S., Jiang Z.H. (2024). A GNSS/SINS fault detection and robust adaptive algorithm based on sliding average smooth bounded layer width. Meas. Sci. Technol..

[26] Geng Y.R., Wang J.L. (2008). Adaptive estimation of multiple fading factors in Kalman filter for navigation applications. GPS Solut..

[27] Miloud H., Abdelouahab H. (2016). Improving mobile robot navigation by combining fuzzy reasoning and virtual obstacle algorithm. J. Intell. Fuzzy Syst..

[28] Abdolkarimi E.S., Mosavi M.R. (2020). Wavelet-adaptive neural subtractive clustering fuzzy inference system to enhance low-cost and high-speed INS/GPS navigation system. GPS Solut..

[29] Taghavifar H., Mardani A. (2013). A knowledge-based Mamdani fuzzy logic prediction of the motion resistance coefficient in a soil bin facility for clay loam soil. Neural Comput. Appl..

